# Decreased levels of regulatory B cells in patients with acute pancreatitis: association with the severity of the disease

**DOI:** 10.18632/oncotarget.23911

**Published:** 2018-01-03

**Authors:** Liannv Qiu, Yonglie Zhou, Qinghua Yu, Junde Yu, Qian Li, Renhua Sun

**Affiliations:** ^1^ Department of Clinical Laboratory, Zhejiang Provincial People's Hospital, People's Hospital of Hangzhou Medical College, Hangzhou, 310004, China; ^2^ Department of Intensive Care Unit, Zhejiang Provincial People's Hospital, People's Hospital of Hangzhou Medical College, Hangzhou, 310004, China

**Keywords:** regulatory b cells, acute pancreatitis, interleukin-10, diagnostic utility

## Abstract

Early stratification of the severity of acute pancreatitis (AP) is clinically important. Regulatory B cells have been found to be associated with disease activity of autoimmune diseases. However, the role of Regulatory B cells in AP remains unknown. We investigate the dynamic longitudinal changes in circulating IL-10-producing B cells (B10) and memory CD19^+^CD24^hi^CD27^hi^ cells in patients with AP to evaluate their prediction utility for AP severity. B10, CD19^+^CD24^hi^CD27^hi^ cells, inflammatory markers and cytokines were detected in patients with AP immediately after admission to the hospital (day 1), then on the third and seventh days. We observed decreases in lymphocytes, CD19^+^, B10, CD19^+^CD24^hi^CD27^hi^ cells and lower mean fluorescence intensity (MFI) of CD80 and CD86 on B10 or CD19^+^CD24^hi^CD27^hi^ cells in patients with AP, especially in those with severe acute pancreatitis (SAP). CD19^+^CD24^hi^CD27^hi^ cells from patients with AP suppressed the cytokine productions of CD4^+^ T cells and CD14^+^ monocytes, but had impaired ability to induce regulatory T cells response. B10 and CD19^+^CD24^hi^CD27^hi^ cells significantly increased in patients with mild acute pancreatitis (MAP) from day 1 to day 7, whereas these indexes remained stable in patients with SAP. B10 or CD19^+^CD24^hi^CD27^hi^ cells were negatively correlated with the severity index (APACHE II score), inflammatory markers (C-reactive protein, CD64 index), and cytokines (IL-6, IL-17, TNF-α). Furthermore, receiver operating characteristic (ROC) curve analysis revealed that B10 and CD19^+^CD24^hi^CD27^hi^ cells could predict the development of SAP. Thus, the detection of B10 and CD19^+^CD24^hi^CD27^hi^ cells may be a practical way to improve the early assessment of AP severity.

## INTRODUCTION

The severity and course of acute pancreatitis (AP) are difficult to evaluate by assessing the first symptoms and clinical signs alone. Although it is mostly self-limiting, approximately 20–30% of all patients will progress to a severe form, which has approximately a 5% mortality rate [1, 2]. An early prediction of AP severity is important in the evaluation of the clinical prognosis and treatment. In a clinical setting, various methods have been used to predict AP severity, including the Acute Physiology and Chronic Health Evaluation (APACHE) II score, Ranson score, and CRP after the first 24 h [3–4]. Recent studies have shown that CD64 index was correlated with disease severity in SAP and may act as a useful marker for the prediction of the development of SAP [5–6]. The expression of HLA-DR on monocytes was highly related to the effector functions of the monocytes. CD14^+^HLA-DR^low/−^ cells, the myeloid-derived suppressor cells with immunosuppressive lymphocyte function, have also been shown to predict the development of organ failure and a fatal outcome [7]. However, these methods in the prediction of AP severity have limitations in the clinical practice. Research has thus focused on the search for novel biochemical markers that can predict AP severity in the initial 24 h following admission.

At present, it is widely accepted that AP is an inflammatory disorder. As an inflammatory process, AP results in excessive leukocyte activation and increased migration of neutrophils to the inflamed area, with a consequent release of pro-inflammatory mediators including interleukins (IL-6, IL-8, IL-15, IL-33, and IL-17), procalcitonin and tumor necrosis factor-a (TNF-α) [8–13]. To maintain immune homeostasis, the body contains regulatory cells which secrete anti-inflammatory factors (e.g., IL-10, transforming growth factor β (TGF-β)) to limit the inflammation process [14]. Normally, the pro- and anti-inflammatory responses are maintained in a state of balance. In SAP, however, this balance is disrupted, and an abnormal activation of inflammatory cells leads to systemic inflammatory response syndrome (SIRS) and multiple organ dysfunction (MODS).

Studies have shown that T helper cells play critical roles in the pathogenesis of AP [15, 16], but the role of regulatory B cells remains unclear. Regulatory B cells are potent negative regulators of inflammation and autoimmune diseases via the release of IL-10 or TGF-β. B10 cells are now recognized as an important component of the immune system. Two B subsets including memory CD19^+^CD24^hi^CD27^hi^ [17] and immature/transitional CD19^+^CD24^hi^CD38^hi^ B cells [18], both of which can secrete IL-10 *in vitro* depending on various stimulations, have been identified in humans. Human CD19^+^CD24^hi^CD38^hi^ cells have been reported to suppress Th1 and Th17 cell differentiation through the production of IL-10 [19]. Carter NA found that, in humans, under conditions of pan-B cell depletion, including regulatory B cell depletion, the inflammatory response will be uncontrolled [20]. The aim of this study was to assess circulating B10 and memory CD19^+^CD24^hi^CD27^hi^ cells among patients with AP of varying severity at the early phase of the disease (first 48 h from the onset of abdominal pain) and to evaluate their diagnostic utility for the prediction of AP severity.

## RESULTS

### Decreased levels of B10 or CD19^+^CD24^hi^CD27^hi^ cells in patients with AP

**Figure 1:**
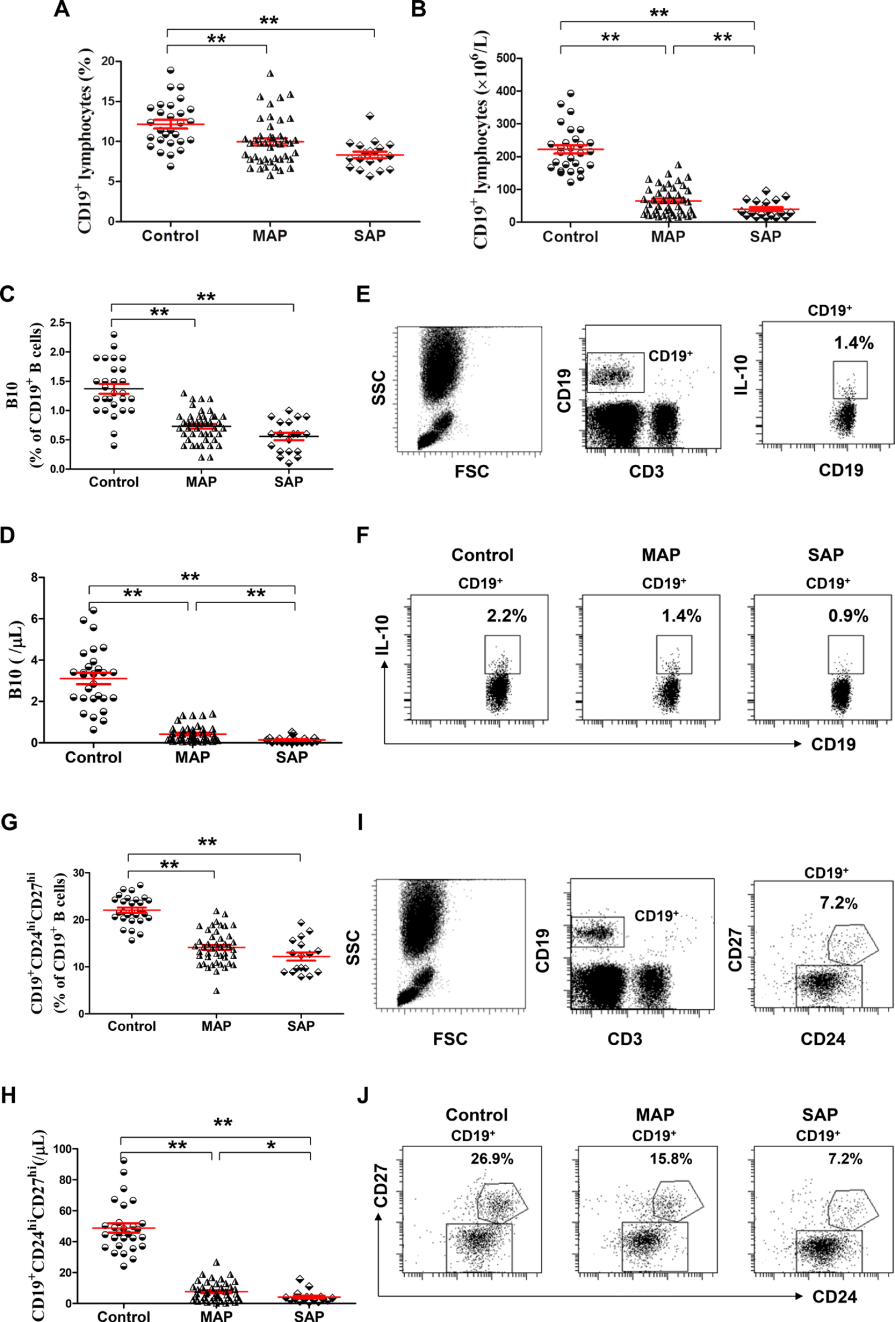
Decreased levels of B10 or CD19^+^CD24^hi^CD27^hi^ cells in patients with AP Graph shows show cumulative data of the frequencies (**A**, **C**) of and numbers (**B**, **D**) of CD19^+^ and B10 cells in healthy individuals (*n* = 21), MAP patients (*n* = 46) and SAP patients (*n* = 17). (**E**) Representative flow cytometry plot depicts the gating strategy for B10 cells. (**F**) Representative dot plots of B10 cells from one healthy individual, one MAP patient and one SAP patient are shown. Graphs show cumulative data of the frequencies (**G**) and numbers (**H**) of circulating CD19^+^CD24^hi^CD27^hi^ cells. (**I**) Representative flow cytometry plot depicts the gating strategy for CD19^+^CD24^hi^CD27^hi^ cells. (**J**) Representative dot plots of CD19^+^CD24^hi^CD27^hi^ cells from one healthy individual, one MAP patient and one SAP patient are shown. ^*^P < 0.05; ^**^P < 0.01.

The numbers of leukocytes were significantly higher but the numbers of lymphocytes were significantly lower in patients with AP on admission than those of healthy individuals (all *P* < 0.001), but no significant difference in the numbers of leukocytes and lymphocytes was observed between patients with MAP and SAP (*P* = 0.0945, *P* = 0.0514, respectively, Table 1). The frequencies and numbers of CD19^+^, B10 and CD19^+^CD24^hi^CD27^hi^ cells in patients with MAP and SAP on admission were below the corresponding frequencies and numbers in healthy individuals (all *P* < 0.001). In addition, the numbers of CD19^+^, B10 and CD19^+^CD24^hi^CD27^hi^ cells in patients with SAP were significantly lower than those in patients with MAP (*P* = 0.0198, *P* = 0.0028, *P* = 0.0313, respectively, Figure 1A–1J).

**Table 1:** Characteristics of the patients with AP and healthy individuals

	MAP	SAP	healthy individuals
Number of patients	46	17	21
Median age in years (range)	56 (21–68)	59 (27–72)	59 (28–70)
Sex (male/female)	27/17	11/8	10/11
Etiology			
Biliary	15	7	
Alcohol	14	9	
Other	10	2	
Unknown	5	1	
Mean APACHE II score	1.27	14.33	
Leukocytes (×109/L)	12.3 (4.2–14.2)	14.6 (8.94–21.0)	5.80 (3.58–7.52)
Lymphocytes (×109/L)	1.06 (0.42–1.56)	0.66 (0.16–1.39)	1.84 (1.24–2.89)
Lipase (U/L)	125.5 (57–189)	311.7 (87–411.2)	17.1 (7.8–34.1)
Amylase(U/L)	107.6 (57.0–164.8) 103.1	(73.3–191.7)	46.1 (35.1–70.3)
Calcium(mmol/L)	2.33 (2.34–2.52)	1.92 (1.67–2.17)	2.31 (2.12–2.49)
CRP (pg/mL)	48.24 (23.9–162.7)	134.8 (71.2–246.1)	2.53 (1.3–8.92)
Pancreatic necrosis		2	

Note: APACHE: Acute Physiology and Chronic Health Evaluation; MAP: Mild acute pancreatitis, SAP: Severe acute pancreatitis. Data are presented as the median (range).

### The lower MFI of CD80 and CD86 on B10 or CD19^+^CD24^hi^CD27^hi^ cells in patients with AP

**Figure 2:**
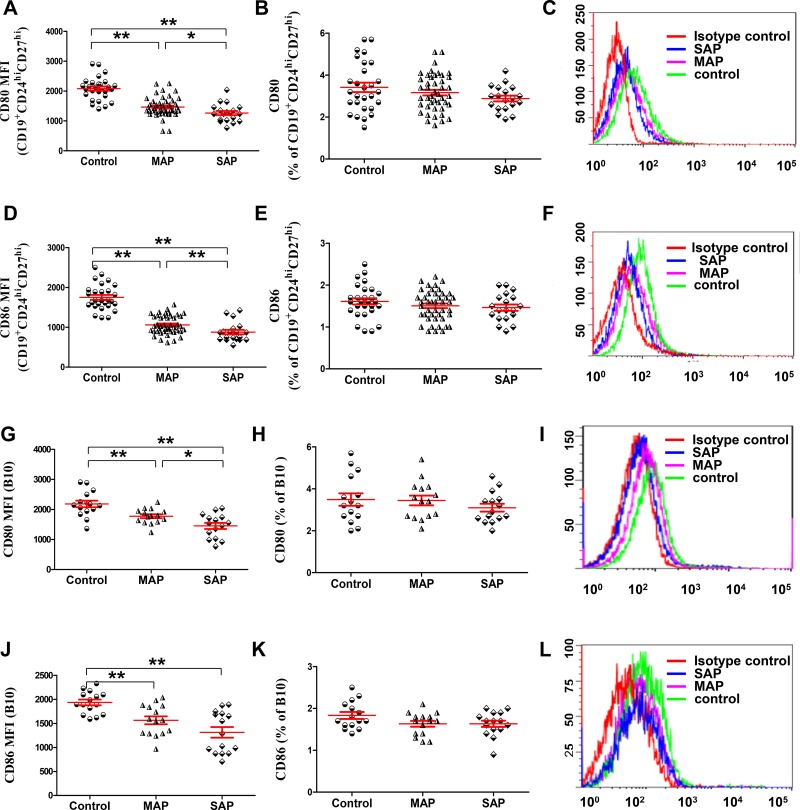
The lower MFI of CD80 and CD86 on B10 and CD19^+^CD24^hi^CD27^hi^ cells in patients with AP Graphs show the cumulative data on the MFI and frequencies of CD80 (**A**, **B**) and CD86 (**D**, **E**) on CD19^+^CD24^hi^CD27^hi^ cells from healthy individuals (*n* = 21), MAP patients (*n* = 46) and 17 SAP patients (*n* = 17). Representative expression of CD80 (**C**) and CD86 (**F**) on CD19^+^CD24^hi^CD27^hi^ cells from one healthy individual, one MAP patient and one SAP patient are shown. Graphs show the cumulative data on the MFI and frequencies of CD80 (**G**, **J**) and CD86 (**H**, **K**) on B10 cells from healthy individuals (*n* = 15), MAP patients (*n* = 15) and SAP patients (*n* = 15). Representative expression of CD80 (**I**) and CD86 (**L**) on B10 cells from one healthy individual, one MAP patient and one SAP patient are shown.^*^P < 0.05, ^**^P < 0.01.

Because B10 and CD19^+^CD24^hi^CD27^hi^ cells were significantly decreased in patients with AP, we investigated the expression of the activation markers CD80 and CD86 by immunofluorescence staining and flow cytometry to determine whether a difference was present in the activation status of B10 or CD19^+^CD24^hi^CD27^hi^ cells between patients with AP and healthy individuals. We noticed that lower MFI of CD80 and CD86 on B10 or CD19^+^CD24^hi^CD27^hi^ cells in patients with MAP and SAP was detected compared with that in healthy individuals (all *P* < 0.001, Figure 2A, 2C, 2D, 2F, 2G, 2I, 2J, 2L); Similarly, the MFI of CD80 and CD86 on B10 or CD19^+^CD24^hi^CD27^hi^ cells in patients with SAP was lower than that in patients with MAP (*P* = 0.029*, P* = 0.0067, *P* = 0.028*, P* < 0.001). However, the frequencies of CD80 and CD86 on B10 or CD19^+^CD24^hi^CD27^hi^ cells were not different between patients with AP and healthy individuals (Figure 2B, 2E, 2H, 2K). Therefore, B10 and CD19^+^CD24^hi^CD27^hi^ cells in patients with AP were in a low activation state.

### CD19^+^CD24^hi^CD27^hi^ cells from AP patients suppress the cytokine productions of CD4^+^ T cells and CD14^+^ monocytes, but have impaired ability to induce tregs response

**Figure 3:**
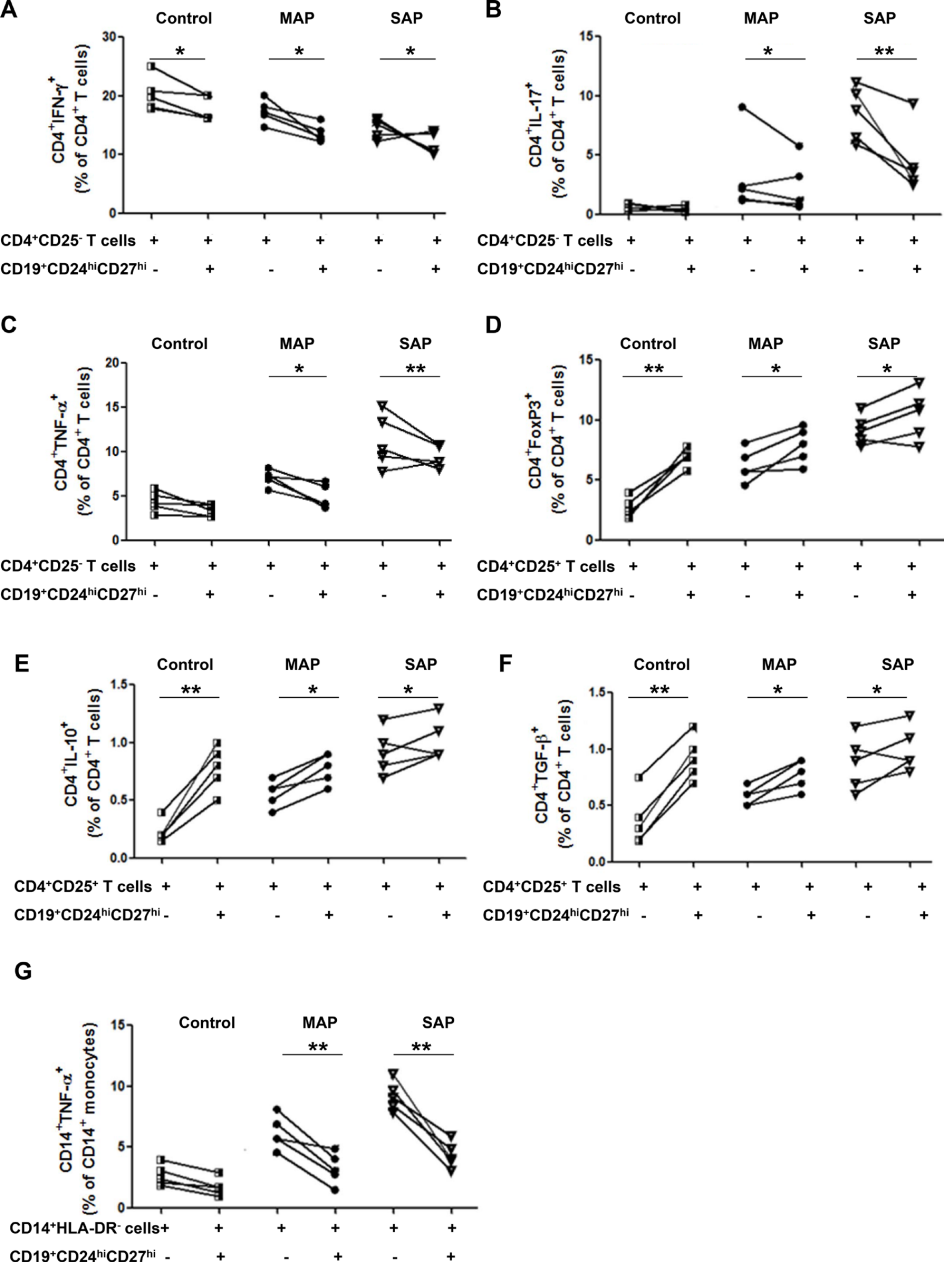
CD19^+^CD24^hi^CD27^hi^ cells from AP patients suppress the cytokine productions of CD4^+^ T cells and CD14^+^monocytes and have impaired ability to induce Tregs response FACS -sorted CD19^+^CD24^hi^CD27^hi^ cells were cultured 1:1 with CD4^+^CD25^−^ T cells, CD4^+^CD25^+^ T cells or CD14^+^HLA-DR^−^monocytes from healthy individuals (*n* = 5) and patients with MAP (*n* = 5) and SAP (*n* = 5). Intracellular levels of IFN-γ, IL-17, TNF-α, IL-10, FoxP3, TGF-β in CD4^+^ T cells and TNF-α in CD14^+^ monocytes were measured by flow cytometry. Graphs show the frequencies of CD4^+^IFN-γ^+^ (**A**), CD4^+^IL-17^+^ (**B**) and CD4^+^TNF-α^+^ (**C**) before or after CD4^+^CD25^−^ T cells coculture with CD19^+^CD24^hi^CD27^hi^ cells. Graphs show the frequencies of CD4^+^FoxP3^+^ (**D**), CD4^+^IL-10^+^ (**E**) and CD4^+^TGF-β^+^ (**F**) before or after CD4^+^CD25^+^ T cells coculture with CD19^+^CD24^hi^CD27^hi^ cells. (**G**) Graphs show the frequencies of CD14^+^TNF-α^+^before or after CD14^+^HLA-DR^−^monocytes coculture with CD19^+^CD24^hi^CD27^hi^ cells. ^*^P < 0.05, ^**^P < 0.01.

To investigate the functional properties of Bregs, CD19^+^CD24^hi^CD27^hi^ cells were sorted from healthy individuals, five patients with MAP and five patients with SAP and co-cultured 1:1 with autologous CD4^+^CD25^−^ T cells in the presence of anti-CD3 and anti-CD28, which has been demonstrated to stimulate T cells activation and up-regulate CD40L expression on T cells: CD40L is necessary for the activation of B cells to exert suppressive function. After 72 h, we observed a slight, but significantly lower percentage of IFN-γ-, IL-17-and TNF-α-producing CD4^+^ T cells when co-cultured with CD19^+^CD24^hi^CD27^hi^ cells compared to CD4^+^CD25^−^ T cells alone from patients with AP (Figure 3A–3C). Similarly, after the co-culture CD14^+^HLA-DR^−^ monocytes with CD19^+^CD24^hi^CD27^hi^ cells from patients with AP, a statistically significant lower percentage of TNF-α-producing CD14^+^ cells was shown when compared to CD14^+^HLA-DR^−^ cells alone (Figure 3G). More obvious suppression the cytokine productions of CD4^+^ T cells and CD14^+^ monocytes was observed in patients with SAP. But no obvious suppression the cytokine productions of CD4^+^ T cells and CD14^+^ monocytes was observed in healthy individuals.

After the co-culture CD4^+^CD25^+^ T cells with CD19^+^CD24^hi^CD27^hi^ cells from patients with AP, a slight elevation of CD4^+^FoxP3^+^ T cells was shown when compared to that in CD4^+^CD25^+^ T cells alone (Figure 3D). We also analyzed the production of IL-10 and TGF-β on CD4^+^CD25^+^ T cells from patients with AP and found that CD19^+^CD24^hi^CD27^hi^ cells increased the production of IL-10 and TGF-β on CD4^+^CD25^+^ T cells in patients with AP and healthy individuals (Figure 3E–3F). However, more obvious enhancement was found in healthy individuals, which suggested that the ability of CD19^+^CD24^hi^CD27^hi^ cells to induce Tregs response was partly impaired.

### Elevated levels of Th17 cells and CD4^+^CD25^hi^FoxP3^+^ cells but decreased levels of Th1 cells in patients with AP

**Figure 4:**
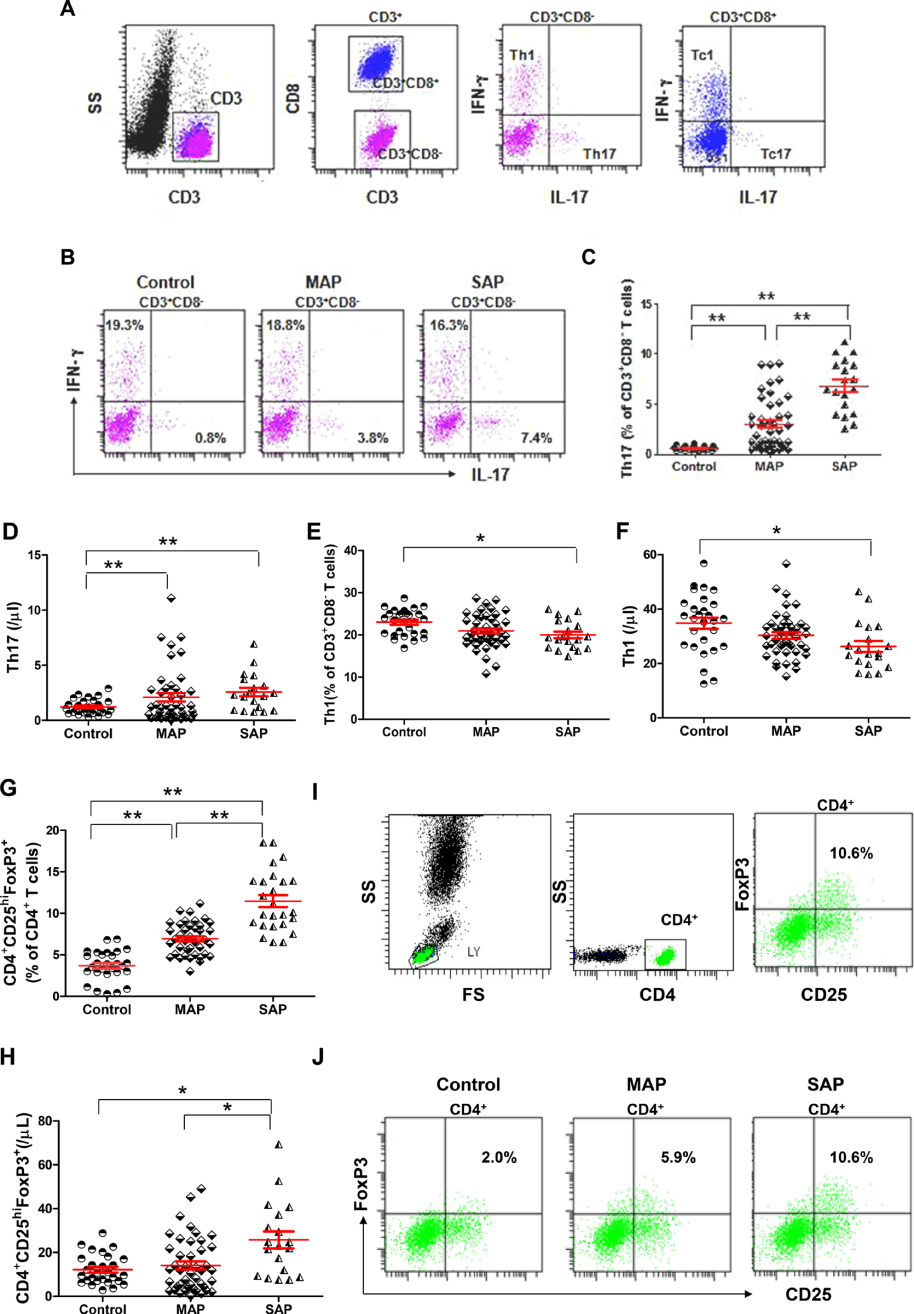
Elevated levels of Th17 cells and CD4^+^CD25^hi^FoxP3^+^ Tregs and decreased levels of Th1 cells in patients with AP (**A**) Representative flow cytometry plot depicts the gating strategy for Th17, Th1, Tc17 and Tc1 cells. (**B**) Representative dot plots of Th1 and Th17 cells from one healthy individual, one MAP patient and one SAP patient are shown. Graphs show cumulative data on the frequencies and numbers of Th17 (**C**–**D**), Th1 cells (**E**–**F**) and CD4^+^CD25^hi^FoxP3^+^ Tregs (**G**-**H**) from healthy individuals (*n* = 21), MAP patients (*n* = 46) and SAP patients (*n* = 17). (**I**) Representative flow cytometry plot depicts the gating strategy for CD4^+^CD25^hi^FoxP3^+^ Tregs. (**J**) Representative dot plots of CD4^+^CD25^hi^FoxP3^+^ Tregs from one healthy individual, one MAP patient and one SAP patient are shown. ^*^P < 0.05, ^**^P < 0.01.

We measured the numbers of Th1 (CD3^+^CD8^−^IFN-γ^+^IL-17^−^) and Th17 (CD3^+^CD8^−^IFN-γ^−^IL-17^+^) cells and Tc1 (CD3^+^CD8^+^IFN-γ^+^IL-17^−^) and Tc17 (CD3^+^CD8^+^IFN-γ^−^IL-17^+^) cells from patients with AP and healthy individuals as described in the Materials and Methods section (Figure 4A). The frequencies and numbers of Th17 cells were significantly higher in patients with MAP and SAP compared with healthy individuals, especially in those with SAP (*P* < 0.001, *P* = 0.0004, *P* < 0.001, Figure 4B–4F). However, the frequencies and numbers of Th1 cells were lower in patients with SAP compared with healthy individuals (*P* = 0.0029*, P* = 0.0064, Figure 4D–4F). No difference in the frequencies and numbers of Tc1 cells and the numbers of Tc17 was observed in patients with AP compared with healthy individuals (Supplementary Figure 1A–1E). Only the frequencies of Tc17 cells were significantly higher in patients with SAP compared with healthy individuals (*P* = 0.0019).

The frequencies of CD4^+^CD25^hi^Foxp3^+^ Tregs were significantly higher in patients with MAP and SAP compared with healthy individuals, especially in those with SAP (all *P* < 0.001). CD4^+^CD25^hi^Foxp3^+^ Tregs were significantly more numerous in patients with SAP compared with patients with MAP and healthy individuals (*P* = 0.0031, *P* = 0.004, Figure 4G–4J).

### CD14^+^HLA-DR^low/−^ cells and CD64 index are elevated in patients with AP compared with healthy individuals

**Figure 5:**
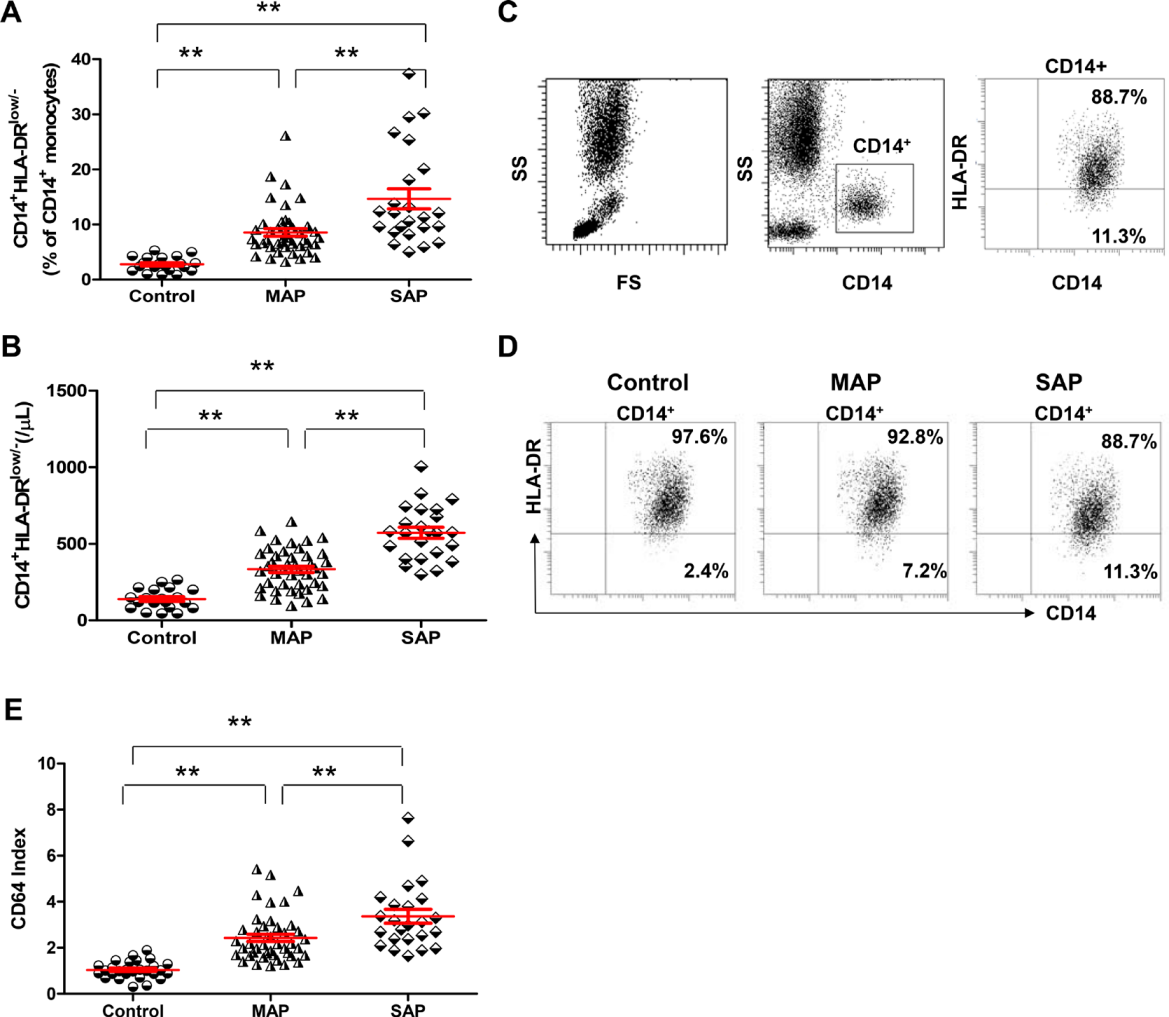
CD14^+^HLA-DR^Low/−^ cells and CD64 index are elevated in patients with AP compared with healthy individuals Graphs show the frequencies (**A**) and numbers (**B**) of CD14^+^HLA-DR^Low/−^ cells from healthy individuals (*n* = 21), MAP patients (*n* = 46) and SAP patients (*n* = 17). (**C**) Representative flow cytometry plot depicts the gating strategy for CD14^+^HLA-DR^Low/−^ cells. (**D**) Representative dot plots of CD14^+^HLA-DR^Low/−^ cells from one healthy individual, one MAP patient and one SAP patient are shown. (**E**) Graphs show CD64 Index from healthy individuals (*n* = 21), MAP patients (*n* = 46) and SAP patients (*n* = 17). ^*^P < 0.05, ^**^P < 0.01.

The frequencies and numbers of CD14^+^HLA-DR^low/−^ cells and CD64 index were significantly increased in patients with AP, especially those with SAP, on admission compared with healthy individuals (all *P* < 0.001, Figure 5A–5E). Interestingly, when we compared MAP patients with healthy individuals, the frequencies and numbers of CD14^+^HLA-DR^low/−^ cells and CD64 index also differed significantly between the 2 groups (*P* < 0.001, *P* < 0.001, *P* = 0.0034).

### Serum cytokines except for IFN-γ are increased in patients with AP

**Figure 6:**
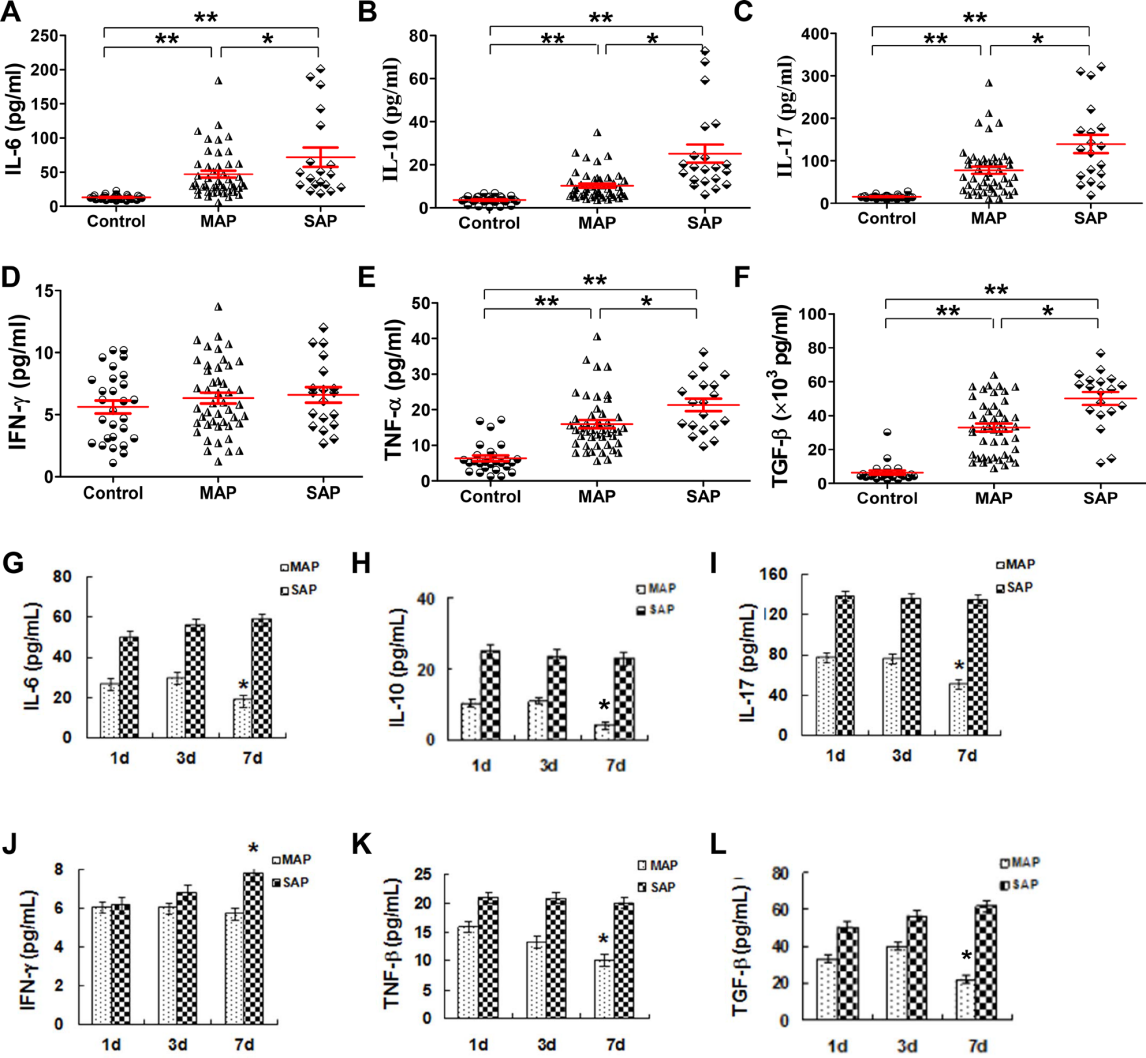
Serum cytokines except for IFN-γ increase in patients with AP Cytokines in serum samples collected from healthy individuals (*n* = 21), MAP patients (*n* = 46) and SAP patients (*n* = 17) were measured by CBA. Graphs show the cumulative data of the levels of IL-6 (**A**), IL-10 (**B**), IL-17 (**C**), IFN-γ (**D**), TNF-α (**E**) and TGF-β (**F**) in healthy individuals (*n* = 21), MAP patients (*n* = 46) and SAP patients (*n* = 17). Graphs show the time course of the changes in the levels of IL-6 (**G**), IL-10 (**H**), IL-17 (**I**), IFN-γ (**J**), TNF-α (**K**) and TGF-β (**L**) from patients with MAP (*n* = 6) and SAP (*n* = 6). ^*^P < 0.05, ^**^P < 0.01.

We know that cytokines play a crucial role in the pathogenesis of AP as they drive the additional inflammatory response, which leads to tissue damages and organ dysfunction. As previously reported [21], our results showed that the serum levels of IL-6, IL-10, IL-17, TNF-α and TGF-β were all increased in patients with MAP and SAP compared with the corresponding levels in healthy individuals (all *P* < 0.001, Figure 6A–6C, E, F); these levels were actually increased along with AP severity, and a significant difference was observed between patients with MAP and SAP (all *P* < 0.05). However, the IFN-γ levels in AP patients were not different from the levels in healthy individuals (Figure 6D). The time course of the change in cytokine levels of six patients with MAP and SAP is shown in Figure 6G–6L. In patients with MAP, the levels of IL-6, IL-10, IL-17, TNF-α and TGF-β decreased from day 1 to day 7 (Figure 6K). In patients with SAP, the levels of all the studied cytokines remained stable except for the levels of IFN-γ which increased over time (Figure 6J).

### Dynamic longitudinal observation of B10 or CD19^+^CD24^hi^CD27^hi^ cells in patients with AP

**Figure 7:**
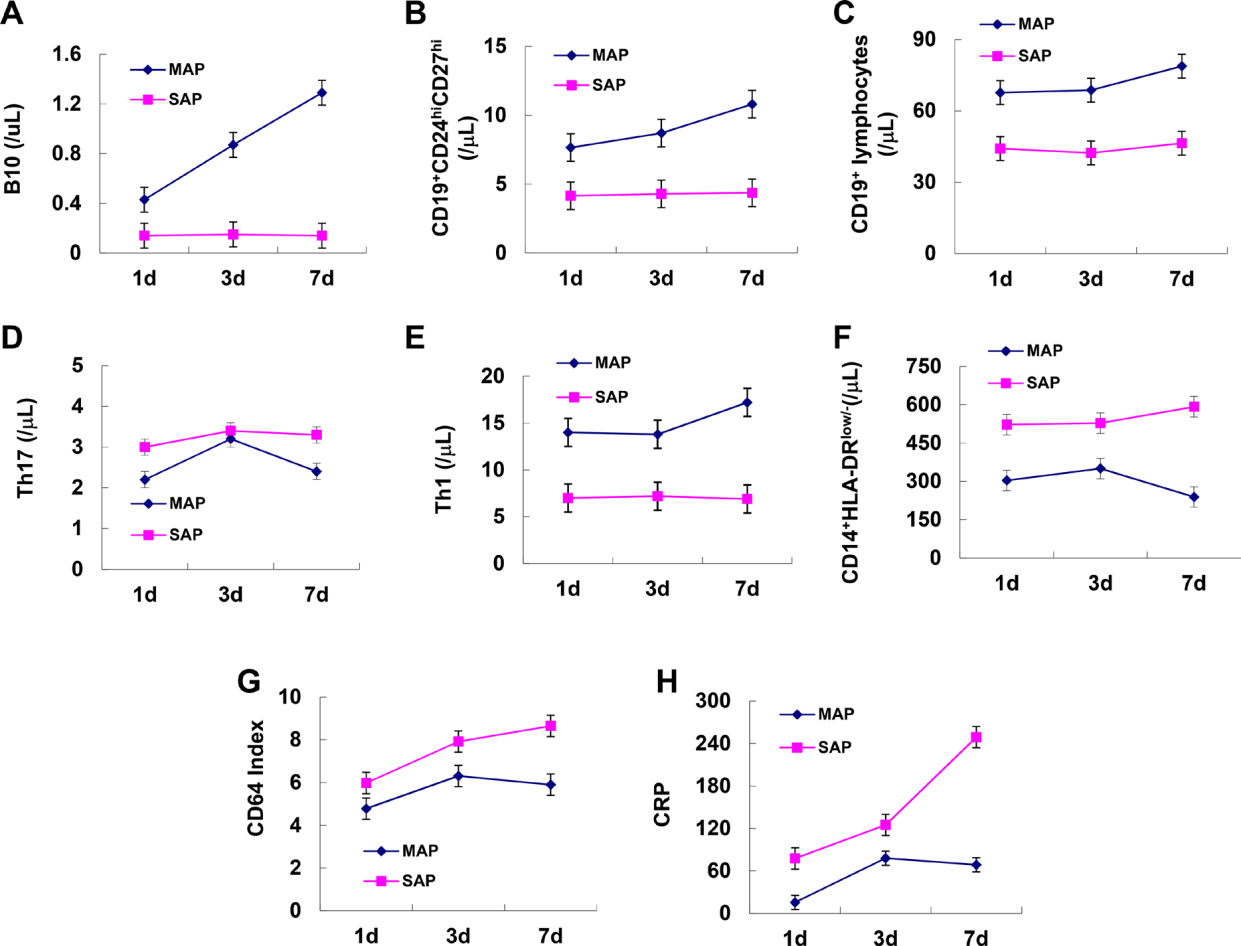
Dynamic longitudinal changes in B10 and CD19^+^CD24^hi^CD27^hi^ cells and inflammatory markers in patients with AP Graphs show dynamic longitudinal changes in the numbers of B10 (**A**), CD19^+^CD24^hi^CD27^hi^ (**B**), CD19^+^ (**C**), Th17 (**D**) Th1 (**E**), CD14^+^HLA-DR^Low/−^ (**F**), CD64 Index (**G**) and CRP (**H**) in patients with MAP (*n* = 6) and SAP (*n* = 6).

To observe the dynamic longitudinal changes in B10, CD19^+^CD24^hi^CD27^hi^ cells, other immune cells and inflammatory markers during the progression of AP, we analyzed six patients with MAP and six patients with SAP from day 1 to day 7. As the disease progressed, B10 and CD19^+^CD24^hi^CD27^hi^ cells were significantly increased, Th17 cells, CD14^+^HLA-DR^low/−^ cells, CD64 index and CRP decreased after an initial rise from day 1 to day 7 in patients with MAP while these indexes remained stable in patients with SAP (Figure 7A, 7B, 7D, 7F, 7G, 7H). CD19^+^ and Th1 cells remained stable over time in both groups (Figure 7C, 7F).

### B10 and CD19^+^CD24^hi^CD27^hi^ cells are correlated with the severity index, inflammatory markers and cytokines in patients with AP

**Figure 8:**
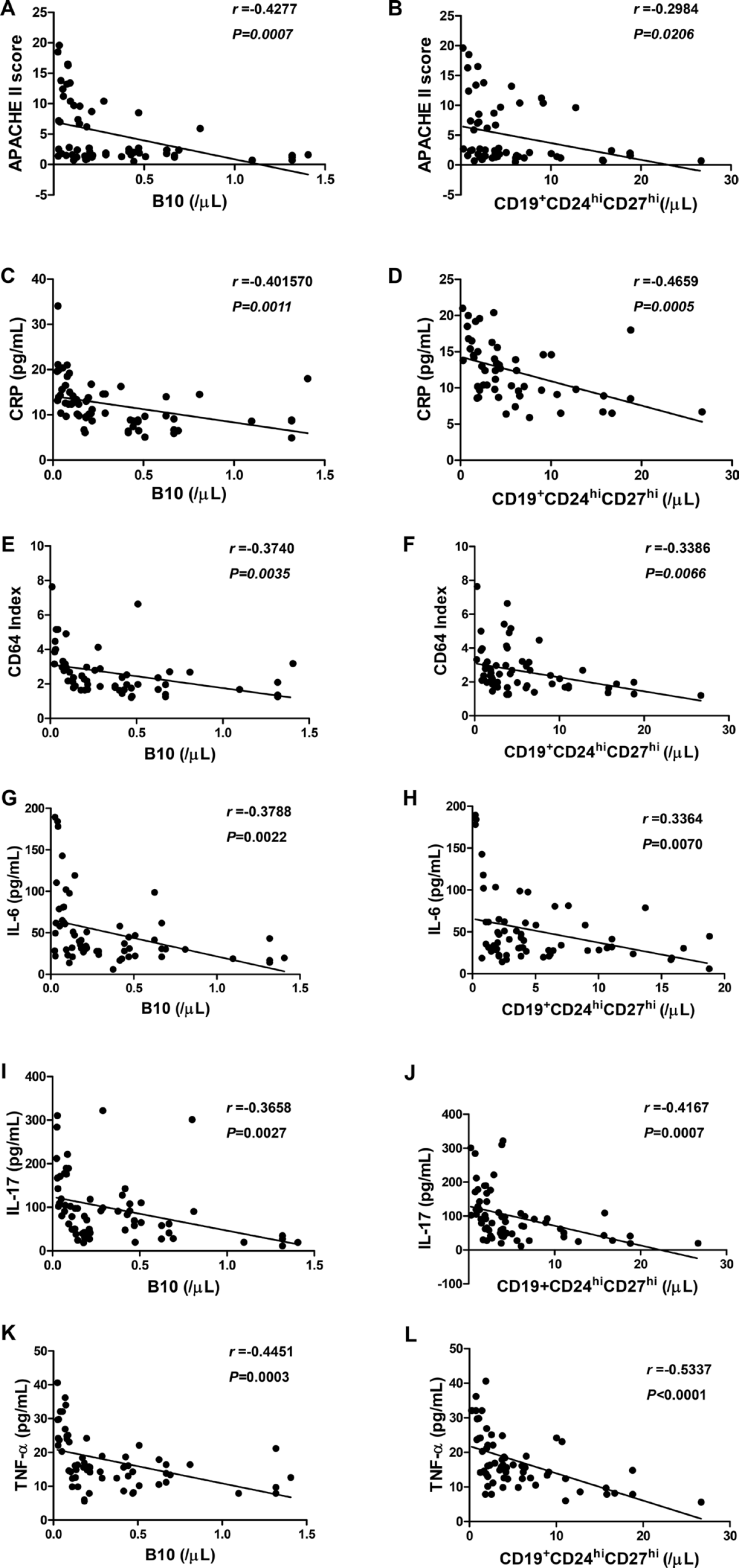
The correlation between B10 or CD19^+^CD24^hi^CD27^hi^ cells and inflammatory markers and serum levels of cytokines in patients with AP Spearman's rank correlation test was performed to compare the respective correlations between B10 or CD19^+^CD24^hi^CD27^hi^ cells and APACHE II score (**A**, **B**), CRP (**C**, **D**), CD64 Index (**E**, **F**), IL-6 (**G**, **H**), IL-17 (**I**, **J**) and TNF-α (**K**, **L**) in patients with AP. R represents a correlation coefficient; a negative R value indicates a negative correlation.

To determine whether the numbers of B10 and CD19^+^CD24^hi^CD27^hi^ cells are inversely correlated with AP severity, we investigated the correlation between B10 or CD19^+^CD24^hi^CD27^hi^ cells with the severity index, inflammatory markers and cytokines in patients with AP on day 3. Interestingly, we found that, to some extent, the numbers of B10 or CD19^+^CD24^hi^CD27^hi^ cells were inversely correlated with APACHE II score, CRP, CD64 index, IL-6, IL-17 and TNF-α levels in patients with AP (Figure 8A–8L). No significant difference was observed between B10 or CD19^+^CD24^hi^CD27^hi^ cells and IL-10, IFN-γ and TGF-β levels at any time point in either group (Supplementary Figure 2A–2F). Taken together, our findings showed that B10 and CD19^+^CD24^hi^CD27^hi^ cells were significantly decreased in correlation with the severity index, inflammatory markers and cytokines, and therefore, the detection of these cells may be useful for the prediction of the development of SAP.

### Diagnostic accuracy analysis of B10 or CD19^+^CD24^hi^CD27^hi^ cells

**Figure 9:**
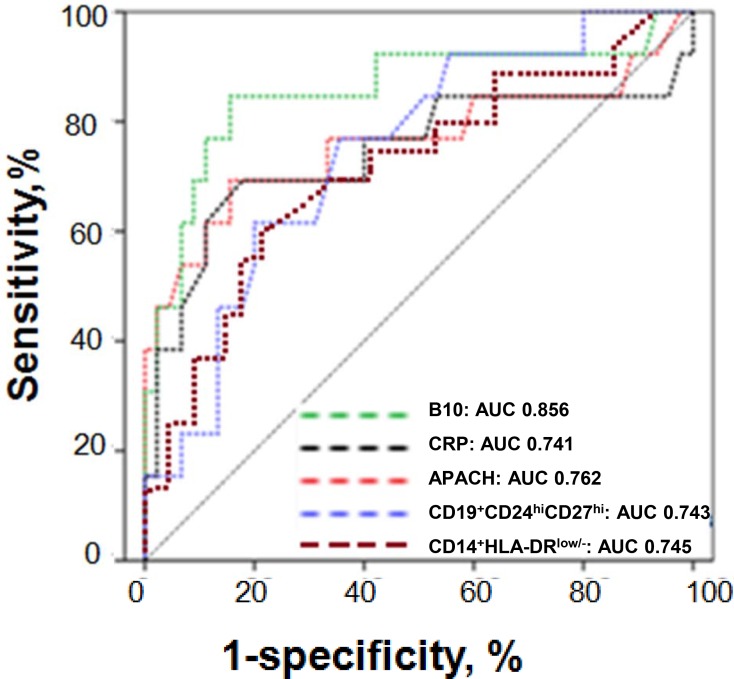
ROC curves for B10 or CD19^+^CD24^hi^CD27^hi^ cells in the prediction of SAP ROC curves for B10 or CD19^+^CD24^hi^CD27^hi^ cells measured within 24 h from the onset of AP in the prediction of SAP in comparison to other laboratory tests associated with AP severity. The selected cut-off values are highlighted, and AUC with 95% confidence intervals and *P*-values for the difference in the AUC from AUC = 0.5 are shown on the graphs. The values of the AUC for each test are shown on the graph. The diagonal line is the line of no-discrimination.

Subsequently, ROC analyses were performed to determine accuracy of B10 or CD19^+^CD24^hi^CD27^hi^ cells for the prediction of the development of SAP. The analyses were initially limited to patients with MAP and SAP immediately after admission to the hospital. The diagnostic utility of the detection of B10 or CD19^+^CD24^hi^CD27^hi^ cells for the prediction of SAP was comparable with APACHE II score, CRP and CD14^+^HLA-DR^low/−^. A B10 count below 0.1366 / μL predicted SAP to a greater degree with a sensitivity of 84% and a specificity of 53%, whereas a count of CD19^+^CD24^hi^CD27^hi^ cells below 4.146 / μL predicted SAP to a greater degree with a sensitivity of 74% and a specificity of 51%. The value of the area under ROC curve (AUC) for B10 was 0.856 [95 % confidence interval (CI) 0.767–0.9372], which was higher than that for CRP (0.741, 95 % CI 0.687–0.895), APACHE II score (0.762, 95 % CI 0.703–0.899), CD19^+^CD24^hi^CD27^hi^ (0.743, 95 % CI 0.679–0.892) and CD14^+^HLA-DR^low/−^(0.745, 95 % CI 0.684–0.897) (Figure 9).

## DISCUSSION

Early stratification of AP severity, especially severe cases, is clinically important for the determination of optimal or intensive treatment to improve outcomes [22]. Clinical scoring systems (APACHE II score and Ranson score) and several serum markers (CRP, procalcitonin, IL-6, and IL-8) have been used to assess the severity of AP [23–24]. However, these approaches have limitations. Conventional severity scores consist of multiple factors (Ranson, 11 factors; and APACHE II, 14 factors) and are complicated, and they may therefore be difficult to accomplish in clinical practice when patients are admitted to a hospital. Taken together, novelpathogenically relevant biomarkers for the early prediction of disease severity are needed.

Immunologic impairment in the early phase of AP may be linked to an increased susceptibility to subsequent infection and the development of septic complications. The phenomenon of immunoparalysis, defined as downregulation of HLA-DR expression on monocytes, and disequilibrium of various helper T cell subsets are usually associated with exaggerated anti-inflammatory responses and high bacterial complications in patients with sepsis, burns, cirrhosis, or AP [14, 15, 25]. These results indicate that immune cells are involved in the pathophysiological process and determine the severity of AP. Regulatory B cells, which are increased in human inflammatory and autoimmune diseases, were previously ascribed with the capacity to suppress autoimmune inflammation, chronic T cell inflammation, and possibly antitumor immunity [26, 27]. Regulatory B cells suppress immune responses via the release of IL-10 or TGF-β, the inhibition of the release of IFN-γ and TNF-α and the induction of Tregs populations as well as interaction with target cells via molecules expressed on the cell surface, such as CD80, CD86 [18, 26–30]. But our study showed a significant reduction in lymphocytes, CD19^+^, B10 and CD19^+^CD24^hi^CD27^hi^ cells in AP patients, which was consistent with the results reported by Qiu et al. [31]. Further, we detected lower MFI of CD80 and CD86 on B10 and CD19^+^CD24^hi^CD27^hi^ cells in patients with AP and CD19^+^CD24^hi^CD27^hi^ cells from AP patients suppressed the cytokine productions of CD4^+^ T cells and CD14^+^ monocytes, but the ability of CD19^+^CD24^hi^CD27^hi^ cells to induce Tregs response were impaired. These results showed that abnormal numbers and functions of B10 and CD19^+^CD24^hi^CD27^hi^ cells may be important contributors to immune dysfunction in AP and may be associated with the degree of organ failure and high mortality in patients with SAP.

It is now established that B lymphocytes contribute directly to enhanced T cell activation and differentiation (e.g., Th1 and Th17) via the production of pro-inflammatory cytokines (such as IL-6 and TNF-α) [32]. Th17 cells and related cytokines (IL-6, IL-17) have been used to assess severity in studies of AP [10, 15, 33–35]. IL-17 promotes the differentiation of B cells into antibody-secreting plasma cells in autoimmune diseases [36]. TNF-α, which is released by many immune cells such as CD14^+^HLA-DR^low/−^ monocytes, is associated with a more severe course of AP, including SIRS and MODS [37]. Just as IL-17 and IL-6 contribute to the differentiation of Th17 cells, IL-10 and TGF-β contribute to the differentiation of regulatory cells [17, 18, 27]. In response to certain stimuli, any B cell could potentially differentiate into a “regulatory B cell,” which can then suppress local inflammation [38]. In our study, serum IL-6, IL-10, IL-17, TNF-α and TGF-β levels were increased in patients with AP at admission and were then decreased on day 7 in patients with MAP, whereas the levels remained stable, with the exception of IFN-γ, in patients with SAP. Function analyses found CD19^+^CD24^hi^CD27^hi^ cells from patients with AP suppressed the release of IFN-γ, TNF-α and IL-17 in CD4^+^CD25^−^ T cells and the release of TNF-α in CD14^+^HLA-DR^−^ cells, but were unable to induce Tregs response in CD4^+^CD25^+^ T cells efficiently. The reduced numbers and abnormal function of B10 and CD19^+^CD24^hi^CD27^hi^ cells promoted the cytokine productions of CD4^+^T cells and CD14^+^ monocytes and induced CD4^+^FoxP3^+^ Tregs expansion, which could further produce a large number of inflammatory factors and cause severe inflammatory damages. Th17 cells further promote the release of pro-inflammatory cytokines such as IL-17, IL-6, and chemokines to expand the inflammatory response, which may eventually cause further immune disorders and lead to SIRS and even death. B10 as well as CD19^+^CD24^hi^CD27^hi^ cells started to increased with time in patients with MAP and suppressed inflammation and restored immune balance, while in patients with SAP they continued to deceased, then aggravated inflammation leads to SIRS and MODS. We conclude that B10 and CD19^+^CD24^hi^CD27^hi^ cells might play a protective role in AP. Further, in our AP patients, B10 and CD19^+^CD24^hi^CD27^hi^ cells reflected the general inflammatory response and predicted the AP severity. However, the identity of the specific cytokines that inhibit the development of B10 and CD19^+^CD24^hi^CD27^hi^ cells requires further study.

To assess the diagnostic value of B10 and CD19^+^CD24^hi^CD27^hi^ cells, we compared the numbers of B10 and CD19^+^CD24^hi^CD27^hi^ cells on day 3 with APACHE II score, CRP, CD64 index and cytokines. Our previous report showed that CD64 index and CD14^+^HLA-DR^low/−^ cells were increased in patients with AP and were highly correlated with APACHE II score [25]. In this study, we noticed that B10 and CD19^+^CD24^hi^CD27^hi^ cells were highly inversely correlated with APACHE II score, CRP, CD64 index and the serum levels of IL-6, IL-17 and TNF-α. Moreover, ROC analyses revealed that B10 or CD19^+^CD24^hi^CD27^hi^ cells predicted the development of SAP, which indicates that B10 and CD19^+^CD24^hi^CD27^hi^ cells may be early markers that can be used to predict the development of SAP.

To the best of our knowledge, this is the first report on the diagnostic utility of B10 and CD19^+^CD24^hi^CD27^h^ cells for the early prediction of AP severity. Our data were based on a limited number of patients, especially those with SAP. For this reason, we were unable to reliably assess the diagnostic utility of B10 and CD19^+^CD24^hi^CD27^hi^ cells for the prediction of SAP. Nonetheless, B10 and CD19^+^CD24^hi^CD27^hi^ cells were significantly decreased in patients with AP, especially in those with SAP, and were negatively associated with the severity of the disease. If this is confirmed in further investigations, the detection of B10 and CD19^+^CD24^hi^CD27^hi^ cells might be a practical way to improve the early assessment of AP severity.

## MATERIALS AND METHODS

### Subjects

The study group comprised 63 patients with AP at Zhejiang's Province People's Hospital and 21 age-matched healthy individuals. AP was diagnosed according to the presence of typical symptoms, such as abdominal pain, and more than three times the normal levels of serum amylase and lipase. All patients fulfilled the criteria for AP and were retrospectively categorized into MAP (*n* = 46) or SAP (*n* = 17) according to the Atlanta classification [39]. The characteristics of the patients are shown in Table 1. Patients who were admitted later than 24 h from the onset of pain due to AP were excluded. Furthermore, patients with chronic pancreatitis, chronic liver diseases (cirrhosis or viral hepatitis), neoplasms of any origin and those treated with immunosuppressants were excluded. Blood samples were immediately obtained from each patient after admission and also on the third and seventh days. All patients received supportive management using intravenous fluid without oral alimentation. Parenteral antibiotics were prescribed for prevention of infection in patients with SAP. An echo-guided or CT-guided aspiration for Gram stain and bacterial culture was performed when infected necrosis or pancreatic abscesses were suspected. Percutaneous drainage or surgical management was performed if infected necrosis or pancreatic abscess was present. All patients were observed until they died or were followed up at the outpatient clinic if they were discharged. None of the patients received drugs affecting immune function.

This study was approved by the Ethics Committee of the Zhejiang's Province People's Hospital. Informed consent was obtained from all individual participants included in the study.

### Cell isolation

PBMCs were isolated from blood samples using a Ficoll-Hypaque density gradient centrifugation method. CD19^+^CD24^hi^CD27^hi^ cells, CD4^+^CD25^−^ T cells (effector T cells), CD4^+^CD25^+^ T cells (Tregs) and CD14^+^HLA-DR^−^ cells from five healthy individuals, five patients with MAP and five patients with SAP were purified using a fluorescence-activated cell sorting (FACS) Aria cell sorter (Becton Dickinson, Palo Alto, CA, USA) based on their expression of CD4, CD25 or CD19, CD24, CD27 and CD14, HLA-DR. Cell purity was confirmed > 95% by flow cytometry.

### Flow cytometric analysis

With respect to the surface marker staining, fresh peripheral blood was tested using the following monoclonal antibodies: APC-CD3 (SK7), FITC-CD19 (J4.119), PerCP-CD27 (L128), PerCP-cy5.5-CD24 (ML5), PE-CD80 (MAB104), PerCP-cy5.5-CD80 (MAB104), PE-CD86 (HA5.2B7), PerCP-cy5.5-CD86 (HA5.2B7), PerCP-cy5.5-CD8 (SK1), PE-CD14 (RMO52), FITC-HLA-DR (Immu-357) and FITC-CD64 (22). After the cells were incubated with the antibodies for 30 min at 4°C in the dark, they were lysed with NH_4_CL solution. Data acquisition and analysis were performed using BD FACSCanto II flow cytometry with FACSDiva software (BD Biosciences, San Diego, CA, USA). The fluorescence intensity of CD64 expression on neutrophils and lymphocytes was measured as the MFI as a linearized value of a log scale. CD64 index is calculated by the ratio of the MFI of the granulocytes to that of the lymphocytes.

For the detection of T helper-secreted intracellular cytokines, peripheral blood mononuclear cells (PBMCs, 2 × 10^6^ cells / mL) were stimulated with phorbol 12-myristate 13-acetate (PMA) (50 ng/mL, Sigma-Aldrich) plus ionomycin (1 mg/mL, Sigma-Aldrich) for 5 hours in the presence of monensin. After membrane staining for CD3 and CD8, the cells were fixed and permeabilized using a Cytofix/Cytoperm kit (BD Biosciences, San Diego, CA, USA) and were stained with PE-conjugated IL-17F (O79-289) and FITC-conjugated IFN-γ (B27). CD3^+^CD8^−^ cells, but not CD3^+^CD4^+^ cells, were defined as T helper cells, due to the downregulation of surface CD4 after stimulation with PMA and ionomycin.

Intracellular IL-10 analysis was performed as previously described [40]. PBMCs were resuspended (2 × 10^6^ cells/mL) in RPMI1640 medium in the presence of CpG (ODN 2006, 10 mg/mL; InvivoGen, San Diego, CA) and CD40 ligand (CD40L, 1 mg/mL; BD Biosciences); PMA (50 ng/mL, sigma), ionomycin (1 mg/mL, sigma), and brefeldin A (5 mg/mL, BioLegend) were added for the last 5 hours of incubation. After membrane staining for CD3 and CD19, the cells were fixed and permeabilized using a Cytofix/Cytoperm kit and stained with a PE-conjugated IL-10 (JES5-19F1).

### Cytokine immunoassays

The serum levels of IL-6, IL-10, IL-17, IFN-γ and TNF-α were determined using BD^TM^ Cytometric Bead Array Human Th1 / Th2 / Th17 kits (BD Bioscience, San Diego, CA, USA). The minimum detectable levels for IL-6, IL-10, IL-17, IFN-γ and TNF-α were 2.4, 4.5, 7.6, 3.7 and 3.8 pg/mL, respectively. TGF-β levels were measured by enzyme-linked immunosorbent assay according to the manufacturer's instructions. All measurements were obtained in duplicate and according to the protocols provided by the manufacturers.

### Function analyses of CD19^+^CD24^hi^CD27^hi^ cells

Isolated CD4^+^CD25^−^ T cells (1.5 × 10^5^) or CD4^+^CD25^+^ T cells (1.5 × 10^5^) were cultured either alone or 1:1 with purified autologous CD19^+^CD24^hi^CD27^hi^ cells (1.5 × 10^5^) in complete RPMI 1640, L-glutamine, and NaHCO3 supplemented with 10% FCS and penicillin/streptomycin (100 U/mL) in 96-well U-bottom plates in the presence of medium or anti-CD3 (1 μg/mL) plus anti-CD28 (1 μg/mL) for 72 h, and with PIB (PMA + ionomycin + brefeldin A) added in the last 5h as described elsewhere. Cells were then surface-stained CD4-APC (4S.B3) permeabilized, stained intracellularly for IFN-γ (FITC, 4S.B3), IL-17F (PE, N49-653), Forkhead box P3 (FoxP3, PE, 259C/C7), IL-10 (PE, JES5-19F1), TGF-β (PE, TW4-9E7) and TNF-α (Alexa Fluo488, MAb11) and analyzed by flow cytometry. Isolated CD14^+^HLA-DR^−^ cells (1.5 × 10^5^) were cultured either alone or 1 : 1 with purified autologous CD19^+^CD24^hi^CD27^hi^ cells (1.5 × 10^5^) in complete RPMI 1640, L-glutamine, and NaHCO3 supplemented with 10% FCS and penicillin/streptomycin (100 U/mL) in 96-well U-bottom plates in the presence of medium for 72 h, and with PIB (PMA + ionomycin+ brefeldin A) added in the last 5 h as described elsewhere. Cells were then surface-stained CD14, permeabilized, stained intracellularly for TNF-α and analyzed by flow cytometry.

### Statistical analysis

Statistical analyses were performed by Student's *t*-test for the comparison of sample means between two groups or by one-way analysis of variance for comparisons of more than two groups. The Pearson product-moment correlation coefficient was used to examine the relationship between two continuous variables. The Wilcoxon matched paired rank test was used for matched paired samples. *P* values < 0.05 were considered to be statistically significant. The data were shown as the median (range) unless otherwise indicated. All analyses were performed using the Statistical Package for the Social Sciences statistical software for Windows, version 11.5 (SPSS Inc., Chicago, IL, USA).

## SUPPLEMENTARY MATERIALS FIGURES


