# Supplementary material for: Decreased levels of regulatory B cells in patients with acute pancreatitis: association with the severity of the disease
Source: Oncotarget. 2018 Jan 3;9(90):36067–82. doi: 10.18632/oncotarget.23911 (PMC6281415; doi:10.18632/oncotarget.23911)
Supplement: Supplementary file 1 [file oncotarget-09-36067-s001.pdf]

Decreased levels of regulatory B cells in patients with acute pancreatitis: association with the severity of the disease

SUPPLEMENTARY MATERIALS

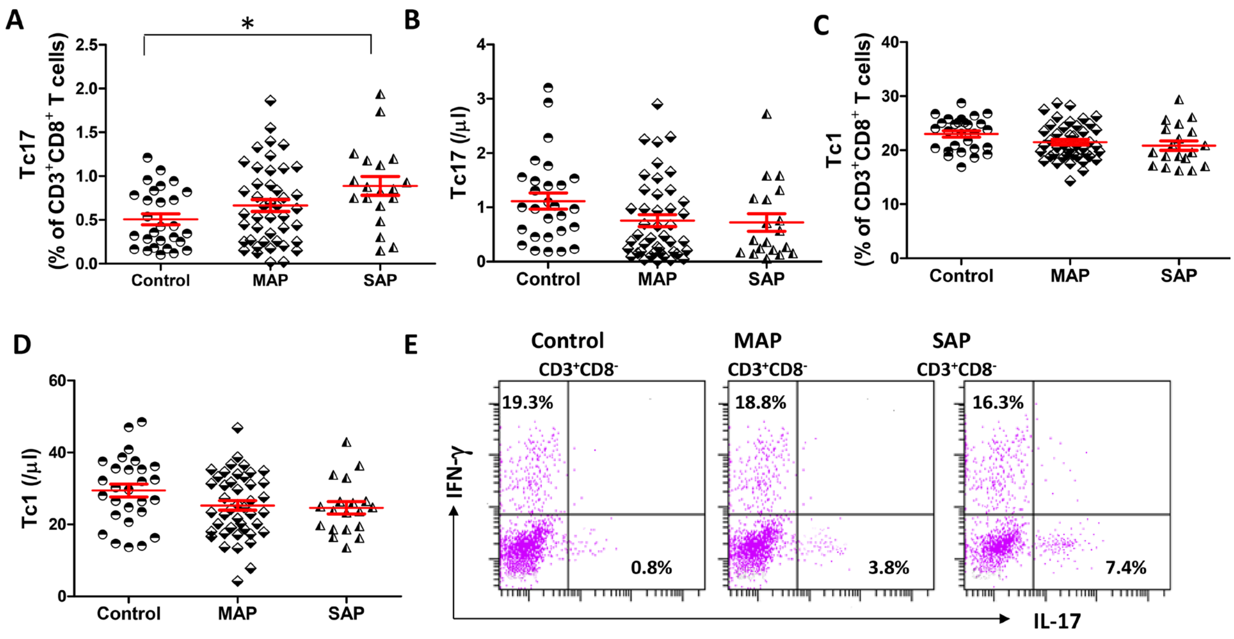

Supplementary Figure 1: Tc17 cells increased in patients with AP. Graphs show cumulative data on the frequencies and numbers of Tc17 cells (**A**, **B**) and Tc1 cells (**C**, **D**) from healthy individuals (n = 21), MAP patients (n = 46) and SAP patients (n = 17). (**E**) Representative dot plots of Tc1 and Tc17 cells from one healthy individual, one MAP patient and one SAP patient are shown. $^{*}P < 0.05$.

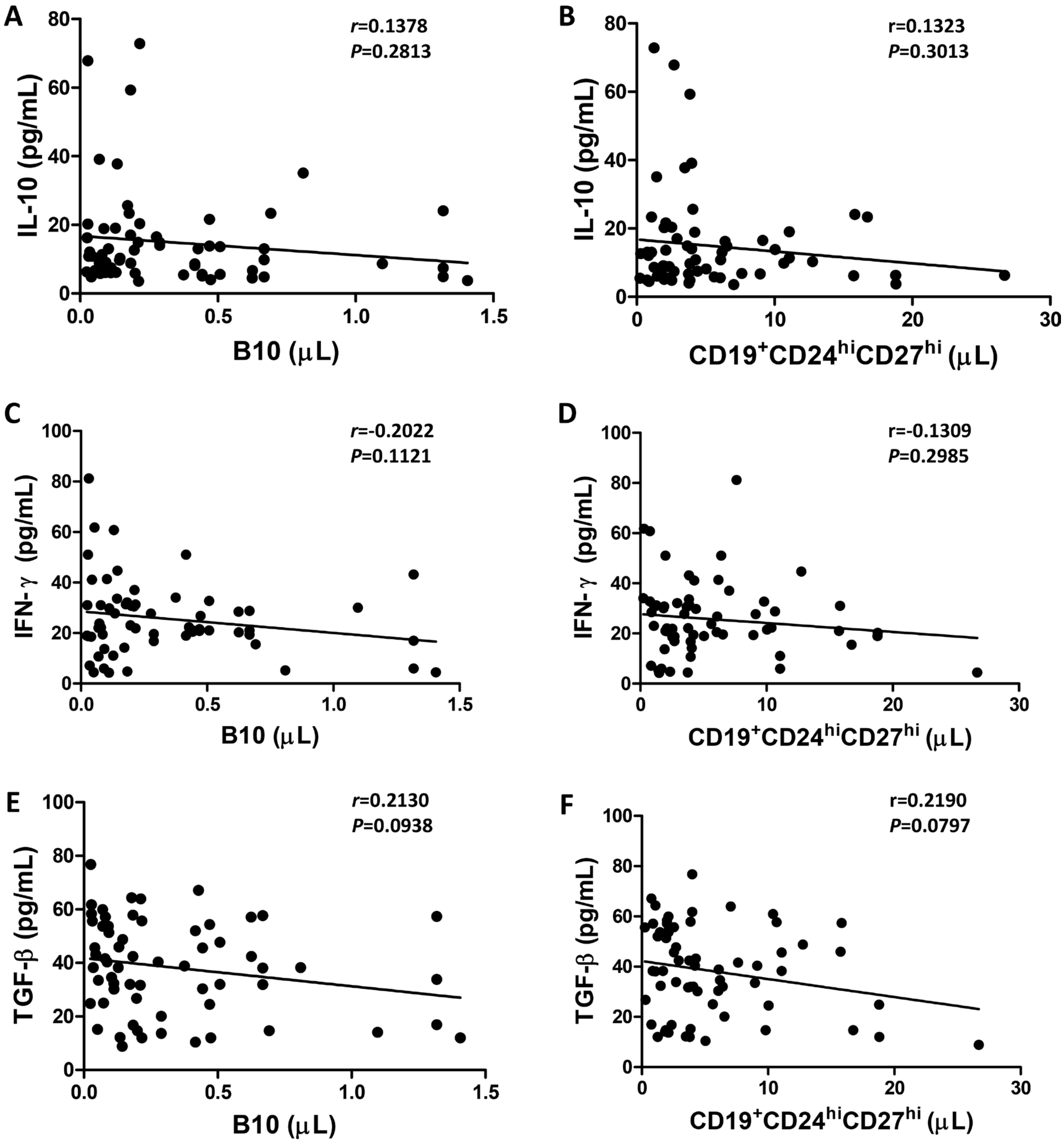

Supplementary Figure 2: The correlation between B10 or CD19+CD24hiCD27hi cells and serum levels of cytokines in patients with AP. Spearman's rank correlation test was performed to compare the respective correlations between B10 or $CD19^{+}CD24^{hi}CD27^{hi}$ and IL-10 (**A**, **B**), IFN-γ (**C**, **D**) and TGF-ß (**E**, **F**) in patients with AP.
